# Anomaly Detection for Sensor Signals Utilizing Deep Learning Autoencoder-Based Neural Networks

**DOI:** 10.3390/bioengineering10040405

**Published:** 2023-03-24

**Authors:** Fatemeh Esmaeili, Erica Cassie, Hong Phan T. Nguyen, Natalie O. V. Plank, Charles P. Unsworth, Alan Wang

**Affiliations:** 1 Department of Engineering Science, University of Auckland, Auckland 1010, New Zealand; fesm704@aucklanduni.ac.nz (F.E.); c.unsworth@auckland.ac.nz (C.P.U.); 2 The MacDiarmid Institute for Advanced Materials and Nanotechnology, Victoria University of Wellington, Wellington 6021, New Zealand; erica.cassie@vuw.ac.nz (E.C.); jenna.nguyen@vuw.ac.nz (H.P.T.N.); natalie.plank@vuw.ac.nz (N.O.V.P.); 3 School of Chemical and Physical Sciences, Victoria University of Wellington, Wellington 6021, New Zealand; 4 Auckland Bioengineering Institute, University of Auckland, Auckland 1010, New Zealand; 5 Center for Medical Imaging, Faculty of Medical and Health Sciences, University of Auckland, Auckland 1010, New Zealand; 6 Centre for Brain Research, University of Auckland, Auckland 1010, New Zealand

**Keywords:** time series anomaly detection, outlier detection, semi-supervised learning, signal processing, autoencoder (AE), vanilla autoencoder, long short-term memory (LSTM), LSTM-based autoencoder, kernel density estimation (KDE)

## Abstract

Anomaly detection is a significant task in sensors’ signal processing since interpreting an abnormal signal can lead to making a high-risk decision in terms of sensors’ applications. Deep learning algorithms are effective tools for anomaly detection due to their capability to address imbalanced datasets. In this study, we took a semi-supervised learning approach, utilizing normal data for training the deep learning neural networks, in order to address the diverse and unknown features of anomalies. We developed autoencoder-based prediction models to automatically detect anomalous data recorded by three electrochemical aptasensors, with variations in the signals’ lengths for particular concentrations, analytes, and bioreceptors. Prediction models employed autoencoder networks and the kernel density estimation (KDE) method for finding the threshold to detect anomalies. Moreover, the autoencoder networks were vanilla, unidirectional long short-term memory (ULSTM), and bidirectional LSTM (BLSTM) autoencoders for the training stage of the prediction models. However, the decision-making was based on the result of these three networks and the integration of vanilla and LSTM networks’ results. The accuracy as a performance metric of anomaly prediction models showed that the performance of vanilla and integrated models were comparable, while the LSTM-based autoencoder models showed the least accuracy. Considering the integrated model of ULSTM and vanilla autoencoder, the accuracy for the dataset with the lengthier signals was approximately 80%, while it was 65% and 40% for the other datasets. The lowest accuracy belonged to the dataset with the least normal data in its dataset. These results demonstrate that the proposed vanilla and integrated models can automatically detect abnormal data when there is sufficient normal data for training the models.

## 1. Introduction

Anomaly detection refers to identifying data instances that have statistically different features from the other available data, considered normal observations [1]. Anomaly detection techniques play increasingly significant roles in various application areas [2], such as medical biomarker detection [3], data mining [4], fraud detection [5], cyber security [6], and industrial quality control [7]. However, establishing an effective anomaly detection technique is demanding due to the scarcity of abnormal observations, diverse patterns of abnormal data, and variable operating situations for data collection. Moreover, accurately labelled datasets of all anomalous data generally are unavailable since identifying abnormal observations normally requires expert knowledge and a huge amount of time. Thus, supervised learning, which needs labelled data, might not be a practical and suitable option for all types of anomaly detection problems.

There are traditional machine learning (ML) algorithms that have been employed for anomaly detection, such as tree-based model [8], density estimation [9], cluster-based method [10], probability estimation [11], and so on. These traditional anomaly detection methods cannot address imbalanced data, unknown features of abnormal data, and unstable operation situations. In this regard, deep learning algorithms have been utilized to address the mentioned issues by applying a semi-supervised strategy [12,13]. More specifically, this technique focuses on abundant normal data for training the anomaly detectors instead of concentrating on abnormal data.

Autoencoder neural network was first developed for dimensionality reduction [14]. Then, it was realized that convolutional-based autoencoders were capable of practical feature extraction from the unlabeled data [15]. Since then, deep learning autoencoder-based neural networks have been gradually exploited for anomaly detection.

For example, Cheng et al. [16] used a residual network (ResNet) autoencoder to determine the abnormality in radar signals. In this work, the structure of the residual network consists of convolutional and long short-term memory (LSTM) layers. The convolutional layers were employed to capture features, and LSTM layers were used to discover the time dependency of data. The accuracy of their model could reach about 85%. Moreover, Othman et al. [17] customized and utilized a ResNet to detect passive seismic data. The proposed residual convolutional neural network was effective for denoising and reconstructing seismic data and did not require any domain knowledge of the noise/signal. The proposed network was capable of automatically detecting abnormal signals in large datasets and real-time monitoring.

Regarding sensor technology, anomaly detection can be considered a principal and important task. The reason is that a sensor can unavoidably be infected by operating situations and sample matrix, or be interfered with on-site contamination. Consequently, this transient signal, affected by these undesirable situations, carries misleading information. Thus, using and interpreting abnormal data in terms of sensors’ application can be resulted in making a high-risk decision. Much research has been carried out regarding sensors’ anomaly detection [18,19].

This paper was motivated by our previous work [20], where we developed an LSTM-based prediction model for classifying the biosensors’ analyte concentration. The outlier detection was addressed by data visualization and labelling with expert knowledge. It means that some of the informative signals might have been lost by only labelling the data when there was uncertainty, even in the behavior of normal data. To the best of our knowledge, there has not been sufficient research on anomaly detection of those time series signals whose sample conditions and analyte concentration have regularly changed during data collection. Thus, there is a necessity for establishing a suitable workflow and proposing an effective anomaly prediction model. Moreover, finding abnormalities and outliers can be regarded as an initial and significant step in solving ML problems, such as classification and regression, since data cleaning is the first step in dealing with an ML problem.

In this study, we exploited autoencoder networks and developed five semi-supervised prediction models to analyze the transient signals from three electrochemical biosensors in order to automate identifying abnormal signals. The automatic anomaly detection models are beneficial and time-saving when confronting a big-size dataset. In addition, deep learning-based anomaly detection models provide a useful understanding of and insight into the behavior and patterns of data. Also, employing deep learning autoencoders provides a useful and essential criterion for anomaly detection in addition to expert knowledge in similar cases where the patterns and features of data are not completely well-known. The reason is that deep learning autoencoders are able to capture the unknown patterns of the data and provide a better understanding of the data. Consequently, a better understanding of the data can prevent losing informative and valuable data; in other words, an automatic anomaly detection method can retrieve and recover the marginal or mislabeled data. Moreover, the output of autoencoder networks is sustainable as these networks can play the role of noise reduction methods. The latent space in these networks acts as a suitable feature extraction method.

**Figure 1:**
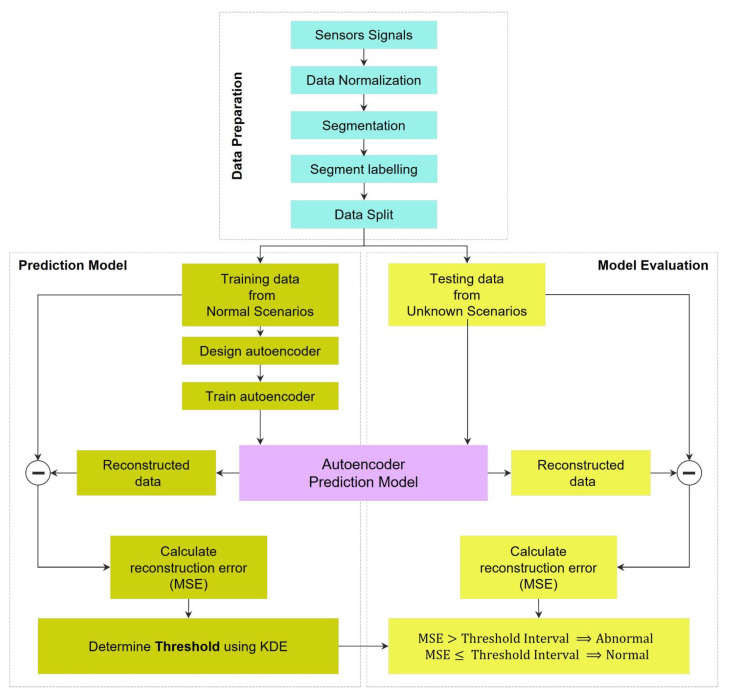
Framework of the presented automated anomaly detection using autoencoder-based neural networks.

The framework of automated semi-supervised anomaly detection methods, depicted in Figure 1, is summarized as follows: First, a data preprocessing method was applied to the time series. After preparing the data for the following stages, the proposed models were trained with normal data. Then, the threshold for anomaly detection was obtained by utilizing reconstructed signals, reconstruction error, and the kernel density estimation (KDE) method. Finally, the test data were used for evaluating the performance of the proposed prediction models.

## 2. Materials and Methods

This section briefly describes the datasets used in this study, then there is a description of the problem. Next, the autoencoder-based anomaly detection technique, which is a vital contribution to this work, is fully proposed. Moreover, it should be noted that all computations and implementation of deep learning algorithms were carried out by MATLAB R2022b.

This workflow can be divided into three main parts: (i) data preparation, (ii) training the network and preparing the prediction model, and (iii) testing and evaluation of the prediction model. These three parts are fully described below.

### 2.1. Datasets Description

A sensor dataset $D=\{X_{1},\ldots ,X_{d},\ldots ,X_{D}\}$ includes a group of signals $X_{d}$ from identical sensing instruments, where subscript *d* is the index of each signal, and *D* is the number of signals in the dataset.

Three datasets were used and analyzed in this work: (1) 35-mer adenosine, (2) 31-mer oestradiol, and (3) 35-mer oestradiol. These datasets consisted of several univariate time-series signals measuring the drain current of three distinctive biosensors. These biosensors were electrochemical carbon nanotube (CNT) field-effect transistor (FET) aptamer-based sensors.

To provide a better insight into the collected signals, the following briefly explains the protocol for drain current measurement. Table 1 represents the main features related to the sensing protocols of the adenosine and oestradiol datasets. It needs to be mentioned that the sensing protocols for 31-mer and 35-mer oestradiol biosensors were exactly identical, but they were slightly different from those of the 35-mer adenosine biosensors.

The drain currents of adenosine and oestradiol sensors were registered in time steps of 1 and 1.081 s with a standard deviation of $5\times 10^{-3}$, respectively. In the initial step, the sensors’ polydimethylsiloxane (PDMS) wells were loaded with specific chemicals. At this step, the sensing response measurements lasted 1000 s and 600 s for adenosine and oestradiol sensors, respectively. Then, as the next step, the analyte solutions were injected into the wells at specific time intervals in successively greater concentrations, considering the total analyte concentrations in the wells before each injection. The time interval of these steps for adenosine and oestradiol sensors were every 500 s and 300 s, respectively. The process of analyte injection resulted in increasing the analyte concentration in the sensors’ wells. The analyte concentrations for adenosine biosensors changed from 1 pM to 10 μM. Likewise, the analyte concentrations for oestradiol biosensors increased from 1 nM to 10 μM.

Since the main focus of this study is to find abnormal signals by utilizing the autoencoder networks, we avoided explaining all characteristics and features of these biosensors. A detailed description of these biosensors’ components and their sensing protocols can be found in our previous article [20]. In addition, comprehensive information on the 35-mer adenosine biosensor, such as fabricating the transistor and functionalizing the aptamer, can be found in [21].

**Figure 2:**
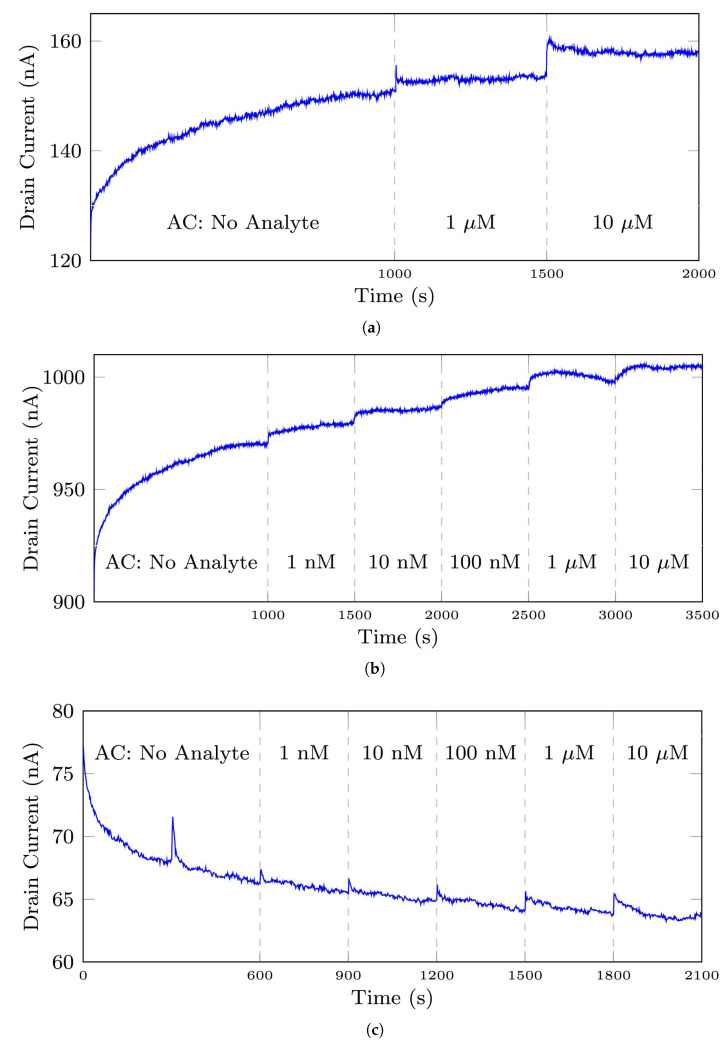

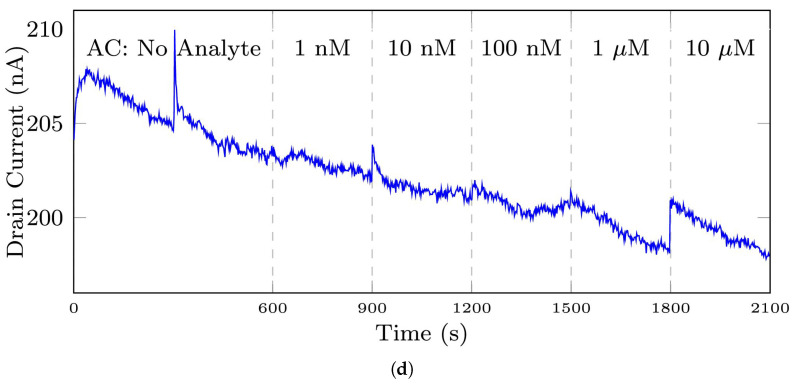
The typical and normal signals measured by biosensors, (**a**) the 35-mer adenosine signal with initial AC of 1 μM, (**b**) the 35-mer adenosine signal with initial AC of 1 nM, (**c**) the 31-mer oestradiol signal, (**d**) the 35-mer oestradiol signal. AC refers to the analyte concentration in this paper.

Figure 2 depicts the typical and normal raw signals of the described datasets. The different analyte concentrations (ACs) in a signal are separated by vertical black dashed lines.

Comparing Figure 2a,b shows that the initial ACs for the 35-mer adenosine biosensors were not necessarily the same. The initial ACs for these experiments were 1 μM and 1 nM, respectively. However, the initial ACs for all the experiments of 31-mer and 35-mer oestradiol biosensors were completely identical, shown in Figure 2c,d, respectively. Table 2 represents the number of entire signals in each dataset.

Further clarification on the notions of an entire signal and a segment is required as we repeatedly use them in this paper. An entire signal refers to all the registered data points of an experiment from its start to its final point, while a segment refers to a section of an entire signal that shows the biosensor’s response to a particular analyte concentration. For instance, in Figure 2a, the entire signal is all data points at t∈[1,2000], while the 1 μM segment is data points at t∈[1001,1500].

### 2.2. Anomaly Description

Abnormal data is determined as a data instance whose behavior and pattern are deviated from the normal and well-defined signals. Detecting abnormal data is essential in machine learning or deep learning problems, since abnormal data contains misleading information and degenerates the performance of the prediction models. The process of finding abnormal data is called anomaly detection. Regarding the time series data, there are three main types of anomalies, which are abnormal time points, time intervals, and time series [22].

Regarding the signals used in this study, several factors affected the signals and generated abnormal data. These factors could be issues and difficulties in fabricating the sensing interface, setbacks in immobilizing the bioreceptor on carbon nanotube surfaces, the failure of the transistor, background noise in the lab, and so on. The data visualization and prior knowledge of the sensor’s behavior were utilized to find the normal signals.

In our previous work [20], the anomalies, called contextual outliers, were detected by data visualization and labelled by the data collectors as normal, marginal, no-sensing signals, and broken transistors. Then, only those signals labelled as normal were kept for making a deep learning prediction model. All those signals that did not slightly conform to the patterns of normal signals were regarded as anomalies, i.e. the marginal, no-sensing, and broken transistor signals.

However, applying merely this strict approach for anomaly detection might be resulted in losing a lot of valuable data and information that could improve the generalization ability of the prediction models. Thus, we utilize an autoencoder-based anomaly detection technique to retrieve the lost informative signals.

**Figure 3:**
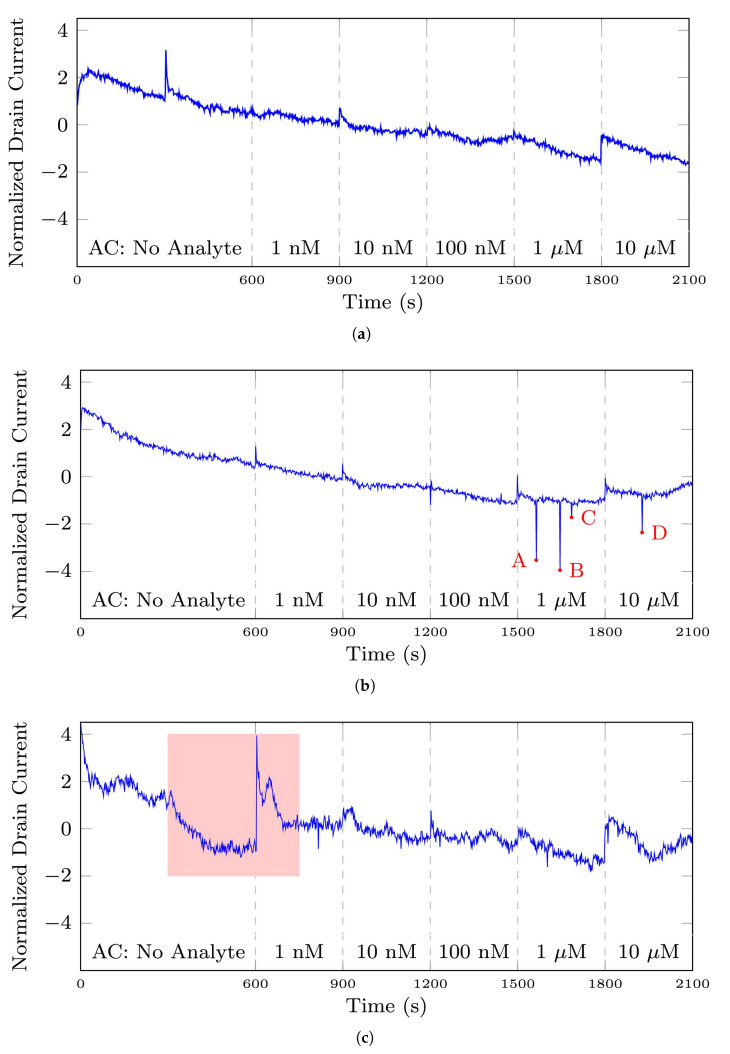

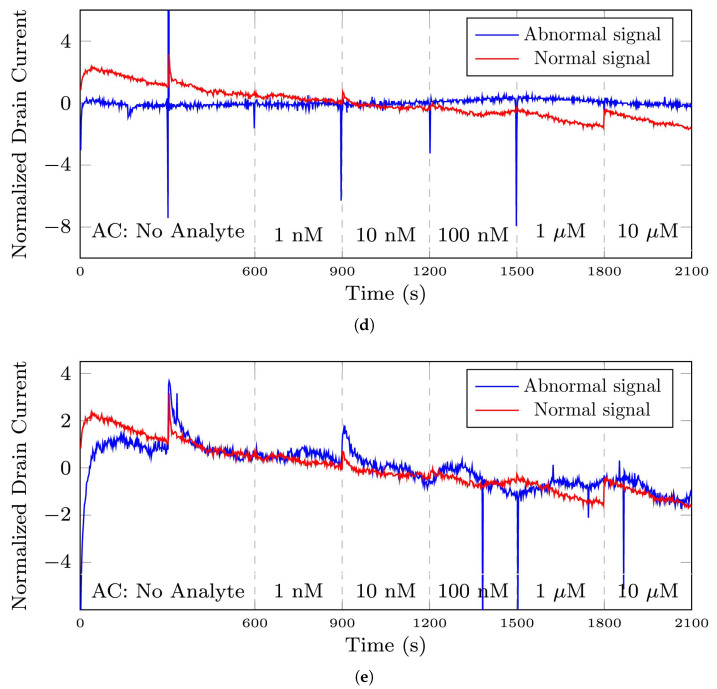
The plots depict the differences between a normal signal and abnormal signals from the 35-mer oestradiol dataset. Note that regarding this dataset, analyte concentration (AC) refers to the volume of oestradiol in the sensor’s well. (**a**) a signal with normal sensing behavior, (**b**) a signal with abnormal time points in A, B, C, and D, (**c**) a signal containing abnormal time interval presented in the pink square area, (**d**) an abnormal time series registered by a broken transistor, (**e**) an abnormal time series that did not show sensing the changes in analyte concentration.

In relation to the described datasets, we explain and clarify the terms normal and anomaly used in this study. Figure 3 compares a normal signal from the 35-mer oestradiol dataset with various abnormal signals in this dataset. Figure 3a is a representative of normal signals in this dataset. All the segments of this signal were labelled as normal.

The main distinguishing features of normal signals were (1) a peak or plateau in the current response after analyte injection and increasing the analyte concentration, and (2) a steady increasing or decreasing current response. Regarding the first feature, it can be seen in Figure 3a that there was a noticeable peak in the drain current when the analyte concentration was increased in the sensor’s PDMS well. The sharpest rise of drain current can be observed at time 1800 s when the level of analyte increased from 1 μM to 10 μM. Also, the other peaks can be seen at other time points of analyte injection, i.e., at t = 600, 900, 1200, 1500. Note that in a well-designed and well-fabricated biosensor, these peaks must be proportional to the analyte concentration. For example, the peak at 1800 s must be larger than the peak at 1500 s.

Regarding the second feature, it can be seen in Figure 2c and Figure 3a that the 31-mer and 35-mer oestradiol sensors’ drain current steadily and smoothly decreased at each time interval of analyte injection. On the other hand, as shown in Figure 2a,b, the drain current of 35-mer adenosine sensors steadily increased over time at each segment after analyte injection.

Figure 3b shows a signal with four abnormal time points. Even though there were four abnormal data points in this signal, this signal was labelled as a normal signal by the experts who collected these signals due to owning two main features of a normal signal. It means that all the segments in this signal were categorized as normal segments, even the 1 μM and 10 μM segments that contain abnormal data points. It needs to be mentioned that these points can be regarded as noises, and the purpose of this study was not noise reduction. However, the autoencoders generally acted as a noise reduction method. These noises were mainly generated due to other devices’ interference in the lab and did not reflect the inefficiency of the biosensors in sensing the analyte.

Figure 3c shows a signal that shows abnormal behavior in a specific time interval, namely from 300 s to 750 s. In such cases, those segments showing abnormal behavior were labeled as anomalies, and the other segments were considered normal segments. For example, in this figure, the No Analyte and 1 nM segments were labeled as anomalies, and the remaining segments were labeled as normal.

Figure 3d,e show abnormal time series that means signal with abnormal behavior throughout the entire signal. The former was recorded with a broken transistor while the latter did not sense the changes in the analyte concentration. All the segments in these signals were labelled as anomalies. It can be seen that these signals did not conform to the red normal signal.

### 2.3. Data Preparation

In this paper, there were four steps in preparing each dataset, including data normalization, segmentation, segment labelling, and data split.

Data normalization aimed to put all the signals in a specific dataset on the same scale and to prevent some features from controlling or influencing other features. The applied normalization method was a Z-score normalization using the mean (μ) and standard deviation (σ) of the entire signal. Consider that $X=[x_{1},\ldots ,x_{i},\ldots ,x_{n}]$ is an entire raw signal with the length of *n*. Then, Equation (1) represents the normalized new signal $\hat{X}$ generated by the described method.

(1) $$X^{Norm}=\left[x_{1}^{Norm},\cdots ,x_{i}^{Norm}=\frac{x_{i}-\mu }{\sigma },\cdots ,x_{n}^{Norm}\right].$$

The effect of the normalization step can be seen by comparing Figure 2d and Figure 3a, as the former is a raw signal instance from the 35-mer oestradiol dataset and the latter is its normalized signal according to Equation (1). Note that the Z-score scaling was applied to the entire signal. Moreover, the reason for applying the normalization step was to put all the signals from each dataset on a similar scale.

Once the normalization was completed, each signal split into its constituent segments. In this paper, a segment refers to a part of a signal with constant analyte concentration from its beginning to its end.

Table 3 shows the number of total segments for each analyte concentration class related to each dataset. Recall that the analyte concentration range for each oestradiol biosensor varied from 1 nM to 10 μM. Thus, the number of entire signals and the number of segments in each class in these datasets is exactly the same. In contrast, the analyte concentration range registered by adenosine biosensors was not the same as each other. Moreover, since there were not sufficient segments for the 100 pM class in the adenosine dataset, this 100 pM class was removed from the dataset. For the same reason, no class number was indicated for this AC class.

In the labelling step, each segment was labelled with its corresponding quality status: Normal or Anomaly. The quality of the signals and segments was determined by the expert who designed the biosensors and experiments and recorded the signals. They assigned the following labels on the signals or segments: normal, marginal, no sense, and broken transistor. In our previous work [20] and in this paper, those signals with marginal, no sense, and broken transistor labels were considered anomalies.

Table 4 represents the total number of normal and anomaly segments regarding each dataset. Recall that the data were labelled by the expert who collected the data. The signals and segments were labelled as normal, marginal, no sense, and broken transistors. Those signals except normal ones were regarded as anomalies.

After labeling the segments, the data were split into training and test sets. The training sets consisted of only normal segments, while the test sets were the remaining part of the total data and consequently a mixture of normal and abnormal segments. The size of the training sets was totally dependent on the available normal segments in each dataset. As only normal data were utilized for training the prediction models, the model can be categorized as a semi-supervised deep learning model.

Regarding the data split, approximately 45% of the 35-mer adenosine dataset was allocated to the training set, while just under 35% of the oestradiol signals were allocated for their training sets. The reason for this inconsistency was related to the insufficient amount of normal segments in oestradiol datasets. By allocating more normal segments to the training sets, their corresponding test sets would be without any normal segments. Consequently, it would not be possible to evaluate the performance of the prediction model on normal segments.

### 2.4. Background of Autoencoder Networks

In this part, we initially demonstrate the theoretical concepts of autoencoder neural networks. After that, the structure of autoencoder networks: (i) vanilla autoencoder and (ii) LSTM-based autoencoder, that was employed in this study will be described.

**Figure 4:**
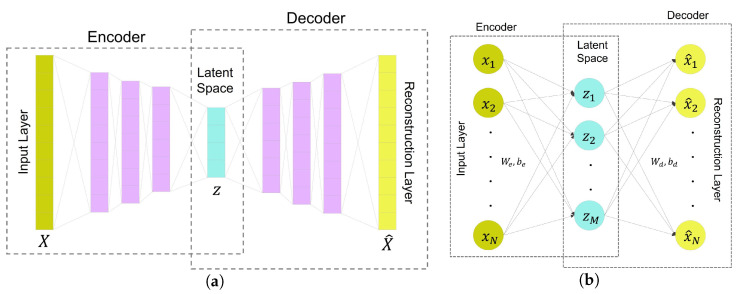
Autoencoder neural networks with their component modules: (**a**) Ilustration of an autoencoder-based network, (**b**) A vanilla autoencoder, Note that the FC layer refers to the fully connected layer.

An autoencoder is an unsupervised neural network with the aim of reconstructing its input to its output and is trained by minimizing the dissimilarity between the input data and its reconstruction output. An autoencoder consists of two modules: an encoder and a decoder [23,24]. Figure 4a depicts the structure of an autoencoder-based network.

When the data (*X*) is fed into the network, the encoder function $f_{\theta_{e}}\;\left(\cdot \right)$ maps the input to the latent space to generate latent value (*Z*). Equation (2) shows the mathematical representation of this mapping.

(2) $$Z=f_{\theta_{e}}\;\left(X\right).$$

The latent space (*Z*) is a nonlinear representation of the input data and retains the underlying features of the input data since the decoder only uses the latent variables to reconstruct the output. Thus, the latent space can be viewed as a good feature extraction of input data [24,25].

Following that, the decoder function $f_{\theta_{d}}\;\left(\cdot \right)$ passes the latent value (*z*) to the output and generates the output value ($\hat{X}$), as shown in Equation (3).

(3) $$\hat{X}=f_{\theta_{d}}\;\left(Z\right).$$

The key objective is to train the mappings $f_{\theta_{e}}\;\left(\cdot \right)$ and $f_{\theta_{d}}\;\left(\cdot \right)$ so that the difference between *X* and $\hat{X}$ can be minimized. The assumption for autoencoder-based anomaly detection is that abnormal data generates larger reconstruction losses. Thus, in most autoencoder-based anomaly detection, reconstruction loss is selected as the anomaly score [24]. Then, by determining a threshold on the training set reconstruction errors, the normal and anomaly data can be predicted [2,26].

It should be noted that the structure of the encoder and decoder can be arbitrary, such as using the LSTM layer or convolutional layer. Also, by designing a deep autoencoder that utilizes many layers of networks for the encoder and decoder parts, the autoencoder is capable of effectively reconstructing complex input data [24,25].

In this study, we use the vanilla autoencoder as the basic network, LSTM-based autoencoders, and utilize the integration of the result of both these networks for decision-making and finding the anomaly signals.

The notable difference between the vanilla autoencoder and the LSTM autoencoder is that the former utilizes the feedforward neural networks in both the encoder and decoder parts, while the encoder and decoder of the latter are built using LSTM layers. The reason for choosing the LSTM autoencoder is that the LSTM supports sequence input data and is suitable for time series forecasting or anomaly detection due to its ability to learn patterns in sequence data. More details of the structure of these networks will be explained in the following parts.

Furthermore, the implementation of all deep learning algorithms was carried out by MATLAB R2022b Deep Learning Toolbox.

#### 2.4.1. Vanilla Autoencoder

A vanilla autoencoder (AE) is a feedforward neural network consisting of only one hidden layer between input and output layers, shown in Figure 4b. In this network, the input and hidden layers form the encoder, and similarly, the hidden layer and output layers build the decoder. The vanilla AE is considered the simplest form of the autoencoder.

In order to have a mathematical representation of these transformations, consider $X\in R^{N}$ as the input to the network, and $f_{e}:R^{N}⟶R^{M}$ and $f_{d}:R^{M}⟶R^{N}$ as the encoder and decoder activation functions, respectively. Equations (4) and (5) show the calculation of hidden layer value ($Z\in R^{M}$) and output value ($\hat{X}\in R^{N}$).

(4) $$Z=f_{e}\;\left(W_{e}\;X\;+b_{e}\;\right),$$

(5) $$\hat{X}=f_{d}\;\left(W_{d}\;Z\;+b_{d}\;\right),$$

where $W_{e}$ and $W_{d}$ are the weight matrices of the encoder and decoder, and in the same order, $b_{e}$ and $b_{d}$ are the bias vectors.

Equation (6) represents the objective in training the vanilla AE; minimizing the loss function L.

(6) $$\underset{W,\;b}{\mathrm{argmin}}\;L\;\left(X\;,\;\hat{X}\;\right),$$

where L can be defined in any form of the difference between the input and its reconstructed output, such as mean square error, $l_{2}$-norm or $l_{1}$-norm [24,25]. Also, *W* and *b* are concatenated representation of weights and biases, i.e., $W=[W_{e};W_{d}]$ and $b=[b_{e};b_{d}]$.

It means that the error, which is the difference between the input and its reconstructed output, is backpropagated through the network in order to update the network’s learnable parameters, i.e., weights and biases.

#### 2.4.2. LSTM Autoencoder

In this part, the structure of LSTM networks will briefly be introduced, and then, the structure of LSTM-based autoencoder will be explained.

The long short-term memory (LSTM) neural network is a generalization of recurrent neural networks (RNNs) that is capable of remembering and learning long-term dependencies of inputs [27,28]. LSTM networks are suitable for analyzing the time series data since it utilizes a gating mechanism for data analysis that is able to capture temporal dependencies of sequential input data during the training of a network. Each LTSM layer is composed of a set of recurrently connected LSTM hidden units, also called LSTM memory cells.

**Figure 5:**
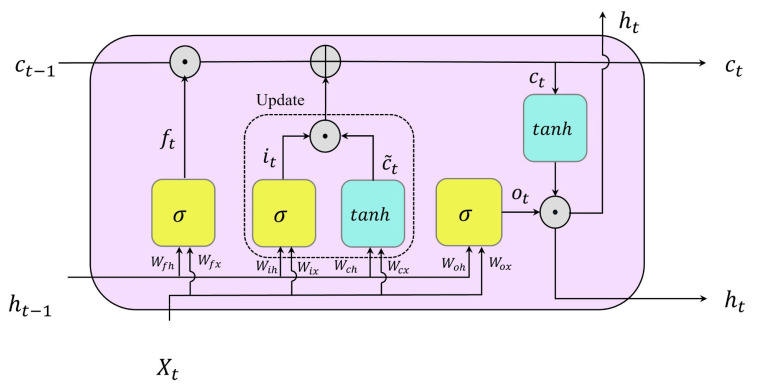
Illustration of an LSTM unit, the building block of LSTM networks. Note that σ and tanh refer to logistic sigmoid and hyperbolic tangent functions, respectively. Moreover, ⊕ and ⊙ point to addition and Hadamard product, also known as element-wise multiplication, respectively.

An LSTM cell, illustrated in Figure 5, includes four parts: the forget gate, the input gate, the output gate, and the cell candidate. In general, the three gates are responsible for selectively controlling and transferring required information into and out of the unit. More specifically, the forget gate selects what information from previous states needs to be erased from the LSTM memory. The input gate and cell candidate are responsible for getting new information and updating the LSTM memory. The input gate controls the values that need to be updated, and the cell candidate adds information to the LSTM memory. The forget gate selects the contributory fraction of information to the output [20,29].

The following equations show the mathematical formulas for calculating $h_{t}$, the output of an LSTM at time step *t*, when the $X_{t}$ is the sequential input to the unit at this time step:

(7) $$f_{t}=\sigma \left(W_{fx}\;X_{t}+W_{fh}\;h_{t-1}+b_{f}\right).$$

(8) $$i_{t}=\sigma \left(W_{ix}\;X_{t}+W_{ih}\;h_{t-1}+b_{i}\right),$$

(9) $${\tilde{c}}_{t}=tanh\left(W_{cx}\;X_{t}+W_{ch}\;h_{t-1}+b_{c}\right),$$

(10) $$c_{t}=f_{t}\times c_{t-1}+i_{t}\times {\tilde{c}}_{t}.$$

(11) $$o_{t}=\sigma \left(W_{ox}\;X_{t}+W_{oh}\;h_{t-1}+b_{o}\right),$$

(12) $$h_{t}=o_{t}\times tanh\left(c_{t}\right),$$

where $W_{fx}$, $W_{ix}$, $W_{ox}$, and $W_{cx}$ refer to the input weight matrices for the forget gate, input gate, output gate, and cell candidate, respectively. Similarly, $W_{fh}$, $W_{ih}$, $W_{oh}$, and $W_{ch}$ are the recurrent weights for the gates and the cell candidate in the same order. Likewise, $b_{f}$, $b_{i}$, $b_{o}$, and $b_{c}$ are their corresponding bias vectors in the same order. Moreover, Equations (13) and (14) mathematically describe sigmoid and tanh functions, respectively.

(13) $$\sigma \left(z\right)=\frac{1}{1+e^{-z}}.$$

(14) $$tanh\left(z\right)=\frac{e^{2z}-1}{e^{2z}+1},$$

**Figure 6:**
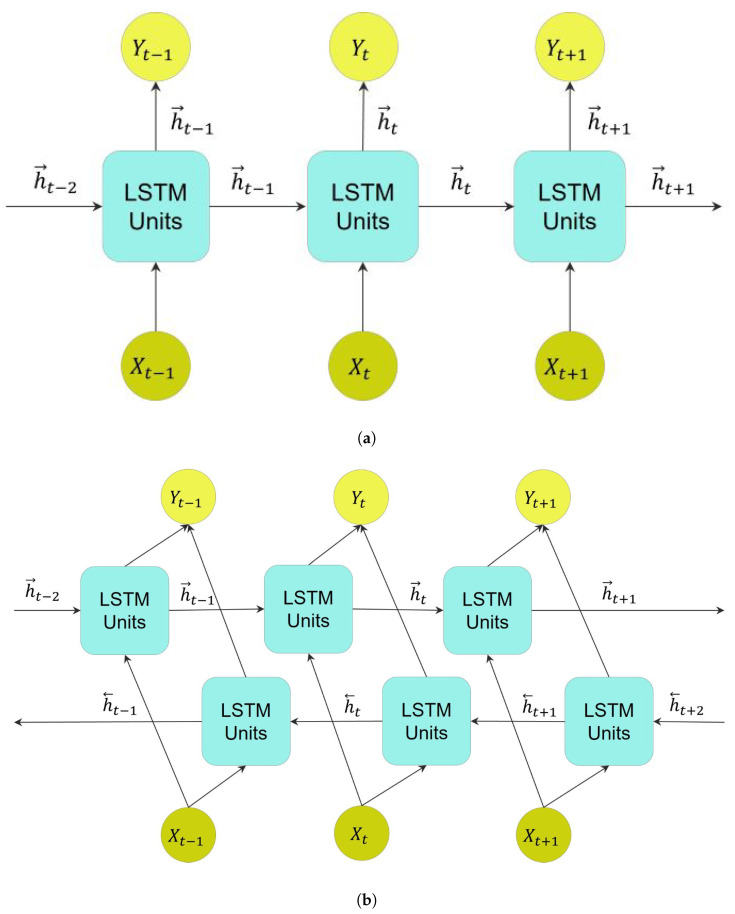
Unfolded LSTM layers in three consecutive time steps depict the flow of information in these layers, where *X* and *Y* refer to the input and output of these layers, respectively. (**a**) A ULSTM layer, where $\vec{h}$ refers to the forward state, positive direction, in this layer. (**b**) A BLSTM layer, where $\vec{h}$ and $\vec{h}$ refer to forward and backward states, respectively.

LSTM layers, consisting of LSTM memory units, are categorized into unidirectional LSTM (ULSTM) and bidirectional LSTM (BLSTM) models [30]. BLSTM is an extension of the ULSTM model composed of two LSTM layers. Figure 6 visualizes both structures, and it can be seen that their main difference of them is in the flow of information direction. As shown in Figure 6a, the flow of information in a ULSTM consists of only forward movements. On the other hand, as shown in Figure 6b, a BLSTM layer consists of LSTM memory units that move the information in both forward and backward directions. In the first round, information at forward units moves in a positive temporal direction. While in the second round, information moves in a negative temporal direction at backward units.

**Figure 7:**
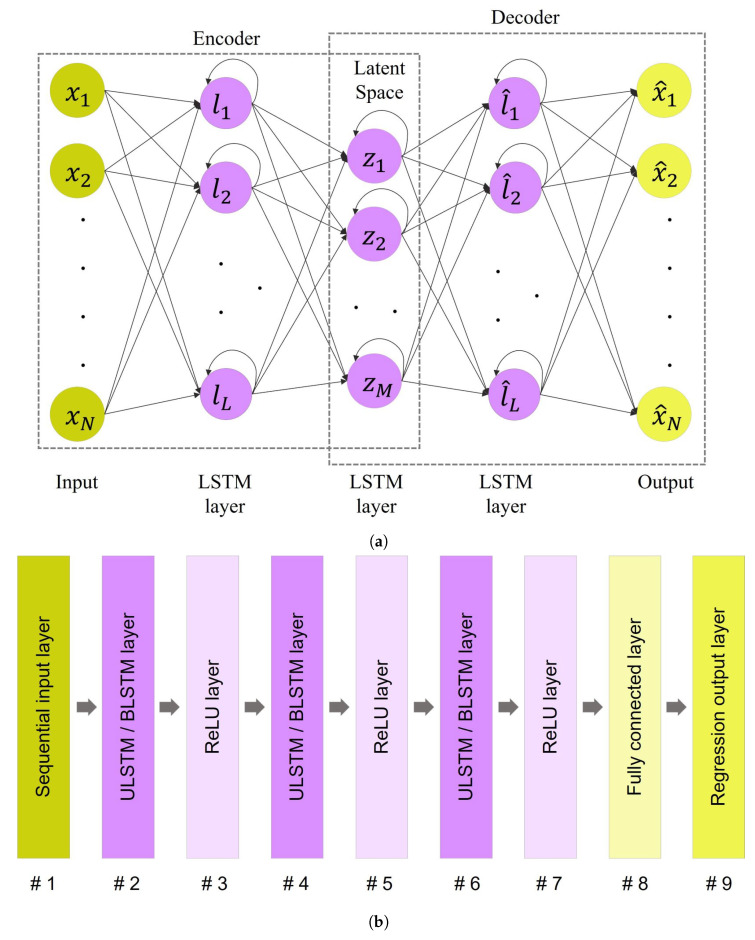
The architecture of LSTM autoencoder: (**a**) A simplified schematic of an LSTM-based autoencoder that both encoder and decoder modules include two LSTM layers. The length of the first and last layers, *N*, is identical to the length of the input sequence. The number of LSTM units in layers 2 and 4 equals *L*; the dimension of these layers. Similarly, there are *M* LSTM units in the third layer. (**b**) Structure of LSTM autoencoder networks. Note that a ULSTM autoencoder and a BLSTM autoencoder differed in their LSTM layers. The former used the unidirectional LSTM layers, while the former used the bidirectional LSTM layers in their structures.

As shown in Figure 7, LSTM-based autoencoder refers to the autoencoder that uses LSTM layers, either ULSTM or BLSTM, in their structures. The capability of LSTM layers to capture patterns in long sequential data makes them suitable for time series anomaly detection [23]. Thus, in order to consider the temporal behavior of the time-series data, we can design an autoencoder based on the LSTM layer.

### 2.5. Prediction Error Calculation

After training the network with normal data, the learnable parameters of the network, such as weights and biases, are determined. At this step, each network’s layer could be regarded as a representation of the input data in different feature spaces [16].

The main idea for anomaly detection is that features of abnormal data are significantly different from normal data [31]. Thus, the trained network produces a detrimental effect on reconstructing the abnormal data. It means that the network’s reconstruction error for normal and abnormal data are quite distinctive [16].

The reconstruction error, also called prediction error, could be computed with a statistical estimator such as mean square error (MSE) [16], mean absolute error (MAE) [32], squared prediction error (SPE) [2], or even absolute value [29].

Since these estimators simply calculate the distance between the original input and the reconstructed output, selecting an error estimator would not affect the final result. Thus, we selected MSE to calculate the reconstruction error. Suppose that $X=[x_{1},x_{2},\ldots ,x_{N}]$ is an input segment and $\hat{X}=\left[{\hat{x}}_{1},{\hat{x}}_{2},\ldots ,{\hat{x}}_{N}\right]$ is its corresponding reconstructed output. Thus, the MSE as the reconstruction error is described in Equation (15):

(15) $$\mathrm{MSE}=\frac{1}{N}\sum_{i=1}^{n}{\left({\hat{x}}_{i}-x_{i}\right)}^{2},$$

### 2.6. Probability Density Estimation of Prediction Error

The next step is to find a threshold as a decision boundary for distinguishing normal data from abnormal data. On the other hand, there is prediction uncertainty in the reconstructed output from the autoencoder network. To address this prediction uncertainty, the reconstructed error, regarded as the prediction error, needs to be determined and modeled [19,33]. There have been various statistical methods for identifying the threshold, such as the maximum likelihood estimation method [23] and the upper quartile [32], in the literature.

To identify the threshold, we used the kernel density estimation (KDE) method to estimate the probability density function of the reconstruction error of normal data in this work, i.e. KDE estimates the density function of the reconstructed errors of normal data in the training set. In addition to estimating the density function, the confidence interval for normal data was estimated by KDE.

KDE refers to a non-parametric test that estimates the density function of samples from an unknown probability distribution [34]. It means that using KDE does not need any assumption and prior knowledge about the data distribution [2,33].

Suppose that $R=\{r_{1},\ldots ,r_{i},\ldots ,r_{m}\}$ is the sample from a population distribution density, H>0 is the smoothing or bandwidth parameter, and K(·) is the kernel function that satisfies the conditions presented in Equation (16):

(16) $$K\left(r\right)>0,\;\mathrm{and}\;\int_{-\infty }^{\infty }K(r)d(r)=1.$$

Then, the estimation of the density function at point *r* is calculated by ρ, presented in Equation (17):

(17) $$\rho \left(r\right)=\frac{1}{mH}\sum_{i=1}^{m}K\left(H^{-\frac{1}{2}}\left(r-r_{i}\right)\right).$$

To interpret the mentioned formula for this paper, the sample *R* from a population distribution was the reconstruction errors from the training set, *m* referred to the number of normal segments in the training set, and the point *r* indicated the reconstruction error.

The critical point for estimating the density function was the bandwidth selection. To address this issue, we used the criterion in [35] to develop an optimal bandwidth $H_{\mathrm{opt}}$, expressed in the following:

(18) $$H_{\mathrm{opt}}\approx 1.06\;\sigma \;m^{-\frac{1}{5}},$$

(19) $$\sigma =\sqrt{\frac{1}{m-1}\sum_{i=1}^{m}{\left(r_{i}-\mu_{r}\right)}^{2}},$$

(20) $$\mu_{r}=\frac{1}{m}\sum_{i=1}^{m}r_{i},$$

where *m* refers to the number of samples. Note that σ and $\mu_{r}$ refer to the standard deviation and mean of samples, respectively.

After fitting a distribution function on the data by KDE, a confidence interval of 90%, $\left[\tau_{l},\tau_{u}\right]$, was set up on that probability distribution.

### 2.7. Anomaly Decision

The final step of making a prediction model in terms of the proposed anomaly detection framework was to find the threshold.

Identifying the confidence interval was equivalent to determining the threshold. It means that the confidence interval played the role of the threshold. To be more specific, the upper bound of this confidence interval was the threshold and acted as the decision boundary for our deep learning anomaly detection. A sample with a prediction error greater than this threshold was regarded as an abnormal sample.

Suppose that *r* is the corresponding prediction error of the sample *x*, ρ(r) is the value of probability density at this point calculated by Equation (17), and $\tau_{u}$ is the upper bound of the 90% confidence interval acquired from the probability density estimation. Thus, the function ζ(x) expresses the mathematical representation of the anomaly decision:

(21) $$\zeta \left(x\right)=\left\{\begin{array}{cc} 0\;\Rightarrow Anomaly & when\;\rho \left(r\right)>\tau_{u}\\ 1\;\Rightarrow Normal & when\;\rho \left(r\right)\leq \tau_{u}. \end{array}\right.$$

Note that the lower bound of the confidence interval, $\tau_{l}$, was ignored for making an anomaly decision. When the reconstruction error of a sample is less than the lower bound of the confidence interval, it means that the original input and the reconstructed output are quite close to each other. Consequently, the output truly mimics the behavior of the input data, which was the desired result and correctly categorized as a normal sample.

### 2.8. Performance Metrics

After developing a prediction model, the performance of the prediction model on unseen data needs to be evaluated. In machine learning terminology, unseen data refers to the test set that has not been used for training the model.

In this study, the prediction models were evaluated using four standard metrics: sensitivity [1,36], precision [1,36], F1-score [1], and overall accuracy [36,37]. At first, each model’s confusion matrix for the test data was generated. Then, the performance metrics were calculated with that confusion matrix. The following equations show formulas for calculating them:

(22) $$\mathrm{Sensitivity}=\mathrm{Recall}=\mathrm{TPR}=\frac{\mathrm{TP}}{\mathrm{TP}\;+\;\mathrm{FN}},$$

(23) $$\mathrm{Precision}=\mathrm{PPV}=\frac{\mathrm{TP}}{\mathrm{TP}\;+\;\mathrm{FP}},$$

(24) $$F1-\mathrm{score}=2\times \frac{\mathrm{Precision}\times \mathrm{Sensitivity}}{\mathrm{Precision}\;+\;\mathrm{Sensitivity}},$$

(25) $$\mathrm{Accuracy}=\frac{\mathrm{TP}\;+\;\mathrm{TN}}{P\;+\;N}.$$

## 3. Results

### 3.1. Structures of Autoencoder Networks

The procedure for making a prediction model for anomaly detection was described in the previous section, including designing the autoencoder-based network, training the network and calculating the reconstruction errors, estimating the probability estimation function, finding the 90% confidence interval and identifying the threshold, and making the anomaly decision.

Thus, the next step after data preprocessing was to design the autoencoder-based networks for training and making deep learning prediction models.

The input layer size for all autoencoder networks was equal to the segment length of each data set. As the output was a reconstruction of the input data, the output layer size needed to be the same as the input size.

Regarding the vanilla autoencoder network, depicted in Figure 4b, we used the built-in network *trainAutoencoder* in MATLAB Deep Learning Toolbox R2022b with 16 hidden units in the hidden layer.

Regarding LSTM autoencoder networks, Figure 7b describes the layer orders and structures of LSTM autoencoder networks used in this study [32]. The number of hidden units in layers # 2 and # 6 was 32, and the number of hidden units in layer # 4 was set to 16. The networks were trained with Adam algorithm [38], a maximum epoch of 50, a minimum batch size of 5, and shuffling the training data every epoch.

As mentioned in the method section, the latent space acts as a feature extraction of the input data. Then, the decoder module only uses these latent features and variables to reconstruct the output. The layers that created latent spaces in the designed networks were the middle layer for the vanilla autoencoder in Figure 4b, and layer # 4 in the LSTM networks in Figure 7b. Thus, The number of hidden units in the layers creating the latent spaces was equal in all networks, set to 16, in order to pass similar features to the decoder modules of the networks.

Note that the other model arguments or hyperparameters for optimizing vanilla and LSTM autoencoders that are not mentioned here were set by MATLAB Deep Learning Toolbox to their default values. For instance, the training algorithm for vanilla autoencoder was set to its default value, scaled conjugate gradient [39].

### 3.2. Experimental Anomaly Decision

The applied steps for anomaly detection are explained one by one in this part.

**Figure 8:**
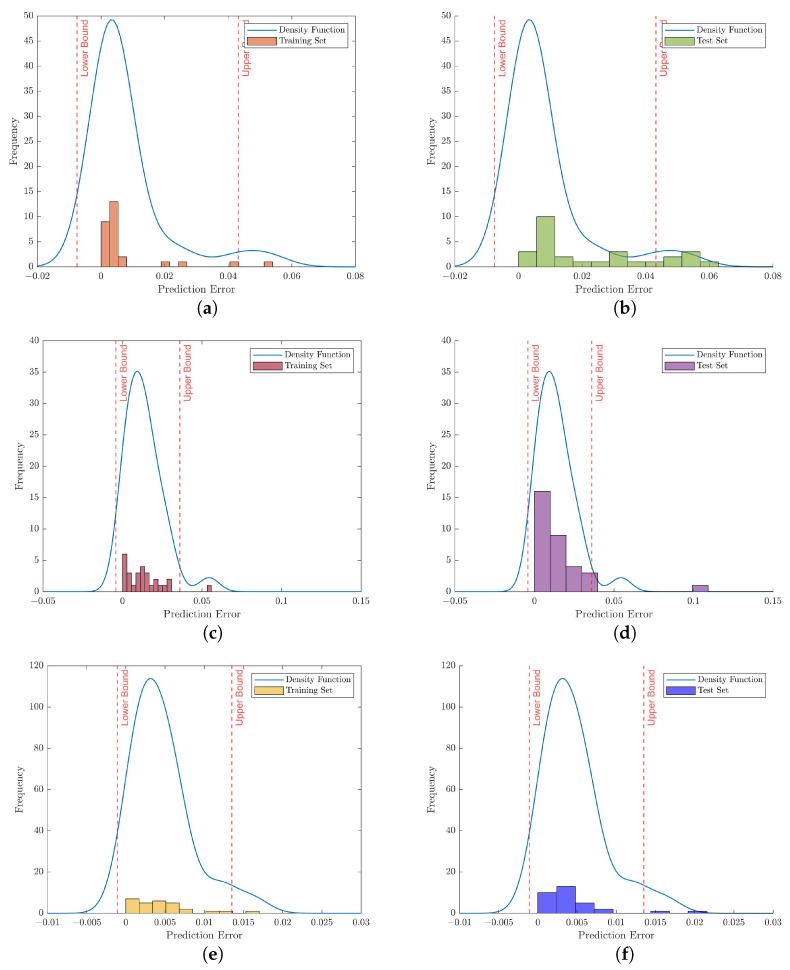
Illustration of anomaly detection based on the prediction error which was estimated by KDE regarding the 35-mer adenosine data set. Note that the histograms on the left column show the reconstruction errors related to the training set with different autoencoder networks. Similarly, the histograms on the right column depict the prediction errors for samples in the test set. (**a**) Trained by vanilla autoencoder, (**b**) Tested by vanilla autoencoder, (**c**) Trained by ULSTM autoencoder, (**d**) Tested by ULSTM network, (**e**) Trained by BLSTM autoencoder, (**f**) Tested by BLSTM network.

- Training the network: when designing the networks was completed, they were trained with the training set containing only the normal segments. Then, each trained network was used, and the corresponding reconstructed outputs for the samples in the training set were generated.
- Calculating the reconstruction errors: then, the MSE expressed in Equation (15) was selected to calculate the reconstruction error for each sample in the training set. The histograms in Figure 8a,c,e represent the distribution of reconstruction errors for 35-mer adenosine training set according to vanilla, ULSTM and BLSTM autoencoders, respectively.
- Estimating density function: in the next step, the density functions for the reconstruction errors were estimated by the KDE method, presented in Equation (17) and the optimal bandwidth in Equation (18). The blue curves in the graphs of Figure 8 represent the estimated density function for the training data.
- Determining the confidence interval: after finding the density function, we used the inverse cumulative distribution function *icdf*, a built-in function in MATLAB, to find the 90% confidence interval for the probability density function. The confidence intervals estimated by KDE corresponding to three autoencoders are surrounded by red dashed lines in the plots of Figure 8.
- Threshold identification: as explained in the materials and methods section, the upper bound of the confidence interval was selected as the threshold to be a decision boundary for detecting abnormal data.
- Anomaly Decision: a sample whose prediction error was greater than its upper bound of the confidence interval was labeled as an anomaly. In Figure 8b,d,f, those samples on the right side of the upper bound red dashed lines were regarded as abnormal samples. On the other hand, the samples on the left side of those red lines were labeled as normal samples.

### 3.3. Visualization of Network-Based Anomaly Detection

Table 5, Table 6 and Table 7 represent examples for data reconstructions and anomaly detection of the explained networks for 35-mer adenosine and 31-mer and 35-mer oestradiol datasets, respectively. The blue lines are the original segment that entered into the network and the red lines show the output of the networks that are the reconstructed segments.

### 3.4. Anomaly Detection Models

In this work, we utilized five anomaly detection models: (1) vanilla autoencoder, (2) ULSTM-based autoencoder, (3) BLSTM-based autoencoder, (4) integration of ULSTM and vanilla autoencoders, and (5) integration of BLSTM and vanilla autoencoders.

The anomaly detection in the first three models was straightforward. The abnormal data on the test set was detected and labeled following the explained pipeline step by step.

However, the anomaly detection procedures for the last two models, the integration of LSTM and vanilla autoencoders, were slightly different, which is explained in detail in the following.

At first, the LSTM model, either ULSTM or BLSTM, was utilized and followed to its final step, which was detecting the abnormal segments in the test set. Then, those abnormal segments were removed from the test set. The remaining data in the test set was labeled according to the vanilla autoencoder model.

To clarify the anomaly detection instruction in the last two models, suppose that *x* was a sample in the test set, $\zeta_{1}\left(x\right)$ and $\zeta_{2}\left(x\right)$ were the segment labels resulting from the LSTM and vanilla networks, respectively. Table 8 represents the final decision of integrating these models with ζ(x).

### 3.5. Performace Evaluation

In the first step of the model’s evaluation, the confusion matrix for each model was generated, shown in Table 9.

**Figure 9:**
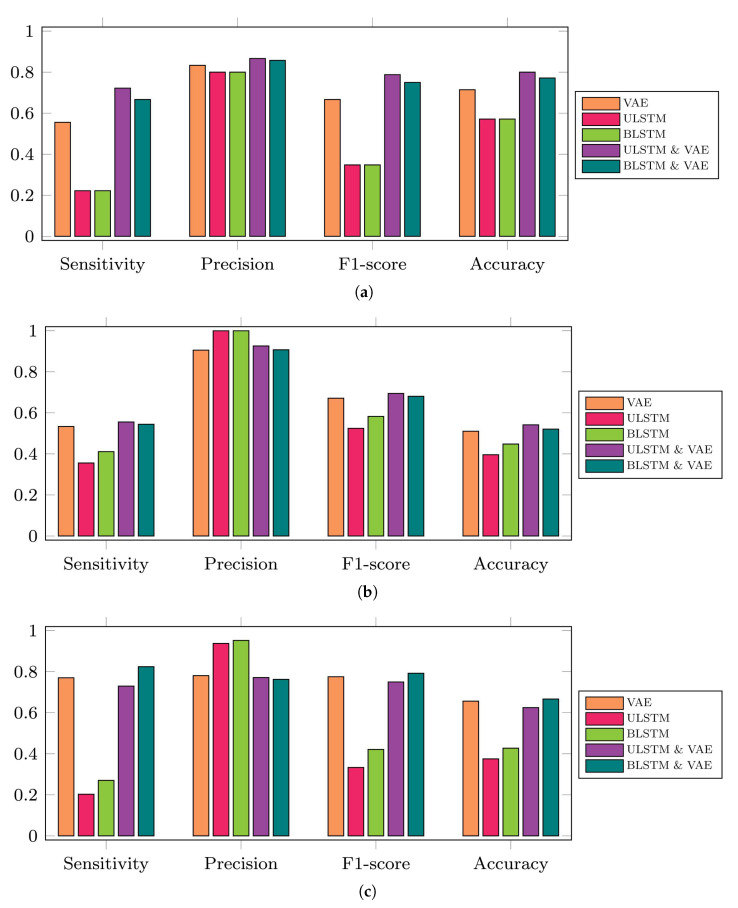
Performance metrics of the five proposed prediction models regarding three available time series datasets for this study. Note that VAE in this figure refers to vanilla autoencoder and it is different from variational autoencoders in some other papers that they also used VAE as the short form of this method. In addition, ULSTM & VAE and BLSTM & VAE refer to the integrated models of LSTM and vanilla autoencoders. (**a**) 35-mer adenosine time series, (**b**) 31-mer oestradiol time series, (**c**) 35-mer oestradiol time series.

The performance and effectiveness of these five prediction models for anomaly detection were assessed by four binary classification metrics, including sensitivity, precision, F1-score, and accuracy. Figure 9 shows the performance of these models for the 35-mer adenosine and the 31-mer and 35-mer oestradiol datasets, respectively.

It can be seen that F1-score and accuracy for vanilla autoencoder and integrated models were quite similar, and these metrics were higher than LSTM autoencoder models in terms of all datasets. F1-score and accuracy of the integrated models were slightly higher than those of the vanilla autoencoder. For example, Figure 9a represents the performance of prediction models on the 35-mer adenosine data. The accuracy for VAE, ULSTM & VAE, and BLSTM & VAE were approximately 71% and 80% and 77%, respectively. On the other hand, the accuracy for ULSTM and BLSTM was 57%.

**Figure 10:**
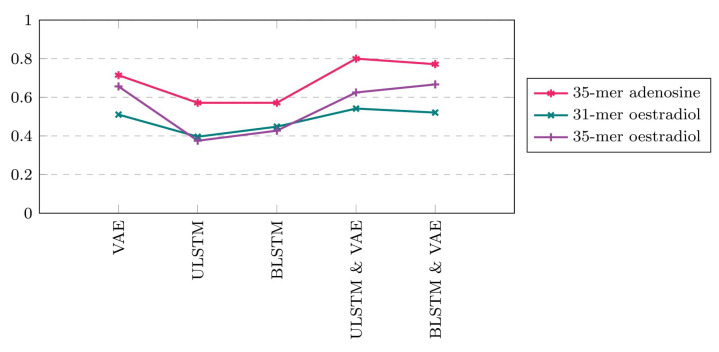
Comparing the accuracy of the models on the available datasets.

Figure 10 compares the anomaly detection models’ accuracy regarding the three datasets. The 35-mer adenosine showed the highest accuracy for all five prediction models compared with two other oestradiol datasets. Considering the ULSTM & VAE model, the accuracy for 35-mer adenosine, the 31-mer, and 35-mer oestradiol datasets was 80%, 54%, and 65%, respectively.

## 4. Discussion

Anomaly detection is a crucial phase in interpreting a sensor’s signal since interpreting an abnormal and misleading signal can lead to making a risky decision. Integrating deep learning algorithms with a semi-supervised approach is a suitable and practical tool for determining anomalies. In addition, this tool is capable of addressing the issues such as varying operation situations and diverse patterns of abnormal data.

Several deep learning-based autoencoders were utilized for time series anomaly detection, data reconstruction, and denoising the data, such as convolutional recurrent autoencoder-based [40] and convolutional-based autoencoder [41,42]. In this study, we successfully developed deep learning autoencoder-based prediction models to detect abnormal signals recorded by three different biosensors automatically. The different features of these sensors were the lengths of time series for particular concentration, their analytes, and their receptors. Prediction models employed autoencoder networks and the KDE method to detect anomalies. The autoencoder networks were trained by the normal data, and then, their reconstruction errors were used as a metric for finding the threshold. After that, the KDE was applied to find the threshold with a confidence interval of 90%.

Moreover, we utilized vanilla, ULSTM, and BLSTM autoencoders for the training stage of the prediction models. However, the decision-making was based on the result of these three networks and the integration of vanilla and LSTM networks’ result. The vanilla autoencoder and integrated autoencoders showed greater performance than the LSTM-based models.

Comparing the datasets, we discovered that the highest accuracy was related to the 35-mer adenosine signals. This result is consistent with our previous work [20] that the highest accuracy of LSTM models for predicting the analyte concentration belonged to this dataset. This result might have occurred for one of two reasons: the length of signals or the chosen criteria for labelling the signals.

Regarding the length of the signal, recall that the sensing responses of 35-mer adenosine segments were registered for 500 s, while this time was 300 s for both oestradiol datasets. In relation to data labelling, more investigation might be needed to label the data. For example, there were several signals registered by sensors that did not seem to display sense. These signals might have been affected and contaminated by other chemicals in the sample. It means the selected normal data might not have been a suitable and comprehensive representation of the normal samples in oestradiol datasets.

On the other hand, deciding on the least accurate dataset was challenging. The reason is that the 35-mer oestradiol signals showed higher performance than the 31-mer oestradiol for the vanilla autoencoder, and both integrated models, while 31-mer oestradiol data performed better for both LSTM-based models. These results challenged our previous results in that ULSTM performed better than BLSTM in the 35-mer oestradiol data for the prediction of analyte concentration [20].

This inconsistency might have occurred due to the slight effect of randomness in deep learning algorithms. Also, it might have happened due to the differences in the structures of LSTM networks designed for classification with the LSTM autoencoder networks designed for anomaly detection. Considering the former possibility of this inconsistency, it can be concluded that the models generally showed better performance in the 35-mer oestradiol dataset than in the 31-mer oestradiol data. The reason for the low accuracy of the anomaly detection model on the 31-mer dataset might have been the insufficient normal data in its dataset.

Even though these methods are not the most advanced models in anomaly detection, applying these methods in the lab is straightforward. The results of these methods might give a better understanding of the data and more critical approaches for biosensor development and data collection. Consequently, using these methods in the lab and in parallel with signal registration might be significantly cost-saving for developing a new biosensor.

In our future work, we can employ more advanced anomaly detection methods, such as using a convolutional neural network (CNN) or U-net and utilizing skip connections to increase convergence speed and accuracy [43] and compare the effect of baseline and simple models with more complex models [44]. Moreover, we can employ a data augmentation technique to extend the length of the available time series datasets by applying deep learning algorithms, such as gated recurrent unit (GRU) and LSTM, and then defining a regression model [45,46]. It means that the length of the time series would be identical by applying this strategy. In the next step, we will design a deep learning model, such as a CNN, that would be able to classify the time series according to their analytes, adenosine or oestradiol. In the next step, the model will be able to predict the analyte concentration [47].

The insufficient data placed limitations on the methods employed in this study. The first limitation was on the applied preprocessing method. The typical data preprocessing method in ML problems is to apply unique and identical changes in both the training and test sets, while we used the Z-score scaling for each time series according to its mean and standard deviation. The reason was that estimating the mean and standard deviation based on the available statistical methods and tests was impossible. The insufficient normal data in test sets exposed the second limitation on the results of this study since we did not confidently compare the effectiveness of the vanilla autoencoder model with the integrated models in recovering the normal data.

## 5. Conclusions

In this paper, we used vanilla autoencoder and LSTM-based autoencoder networks to build prediction models for discovering the abnormal signals of three similar electrochemical aptamer-based sensors in three different datasets: 35-mer adenosine, 31-mer and 35-mer oestradiol datasets. These drain current signals measured the sensing response of the sensors, while there was a successive increment, from 1 nM to 10 μM, in the analyte concentration of the laboratory samples. The principal objective of signal processing and time series analysis was to automate anomaly detection and use the benefits of deep learning networks to gain a deeper and more useful understanding of the time series signals.

The workflow for making the prediction models was based on training the autoencoder networks with normal data and calculating the prediction errors; reconstruction errors. Then, KDE was applied to determine a threshold decision boundary for recognizing the anomaly. We developed five prediction models to find the most accurate and robust models for the available datasets. Three of these prediction models simply used the mentioned networks for training; vanilla, ULSTM, and BLSTM autoencoders. While decision-making step on the other two models was based on the integration of the result of (i) trained ULSTM and vanilla networks or (ii) trained BLSTM and vanilla networks.

The prediction models utilizing the vanilla autoencoder and those integrations of LSTM and vanilla autoencoders were proven to be highly effective for anomaly detection of time series data. In addition, the result suggests that using a more complex deep learning network, such as LSTM autoencoders, does not necessarily create a more effective prediction model. Integrating different deep learning algorithms can increase prediction models’ robustness, accuracy, and performance.

## Figures and Tables

**Table 1:** Main features of sensing protocols for drain current measurements of the adenosine and oestradiol time-series signals. The information related to the 31-mer and 35-mer oestradiol biosensors is merged into one column due to the identical sensing protocols for these biosensors.

Sensing Protocols	Adenosine Biosensor	Oestradil Biosensors
Analyte	adenosine	oestradiol
Measurement time interval	1 s	1.081 s with std $5\times 10^{-3}$
Initial analyte load time	1000 s	600 s
Time interval of analyte injection	500 s	300 s
Variation of analyte concentration	1 pM–10 μM	1 nM–10 μM

**Table 2:** The number of entire signals in available datasets.

Dataset ID	Dataset Name	Total Signals
1	35-mer Adenosine	15
2	31-mer Oestradiol	24
3	35-mer Oestradiol	24

**Table 3:** The number of available segments for each dataset, classified based on their analyte concentration range.

Class ID	Analyte Concentration	Dataset ID		
		1	2	3
1	No Analyte	15	24	24
-	100 pM	1	0	0
2	1 nM	5	24	24
3	10 nM	7	24	24
4	100 nM	9	24	24
5	1 μM	12	24	24
6	10 μM	15	24	24
Total Segments		63	144	144

**Table 4:** Total number of normal and abnormal segments for the available datasets.

Dataset ID	Dataset Name	Total Normal Segments	Total Abnormal Segments
1	35-mer Adenosine	45	18
2	31-mer Oestradiol	54	90
3	35-mer Oestradiol	70	74

**Table 5:** Visualization of anomaly detection for 35-mer adenosine dataset. This table shows original and reconstructed segments created by designed autoencoder networks. The blue curves were the original time series, and the red curves were the reconstructed time series.

Actual Class	Predicted Class	Vanilla AE	ULSTM AE	BLSTM AE
Normal	Normal	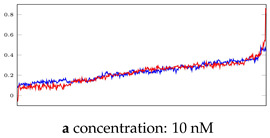	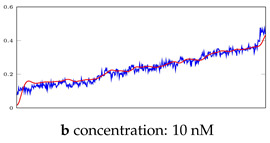	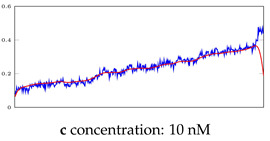
Normal	Anomaly	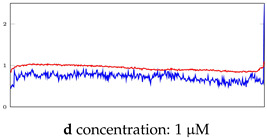	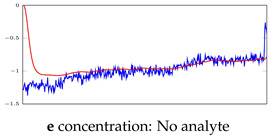	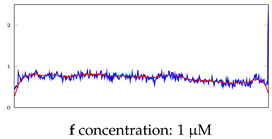
Anomaly	Normal	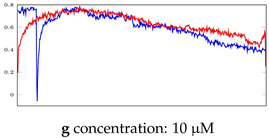	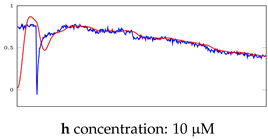	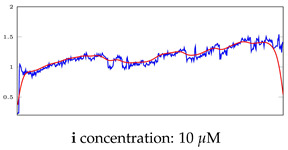
Anomaly	Anomaly	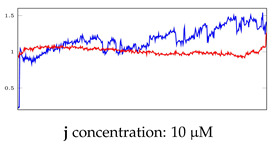	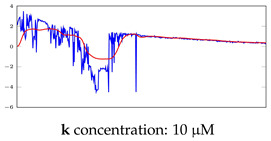	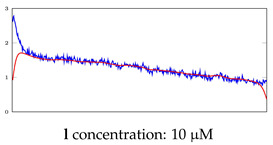

**Table 6:** Visualization of anomaly detection for 31-mer oestradiol dataset. This table shows original and reconstructed segments created by designed autoencoder networks. The blue curves were the original time series, and the red curves were the reconstructed time series.

Actual Class	Predicted Class	Vanilla AE	ULSTM AE	BLSTM AE
Normal	Normal	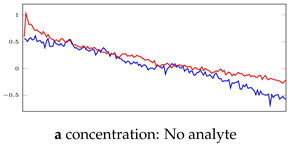	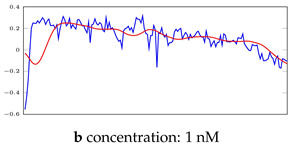	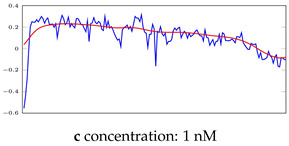
Normal	Anomaly	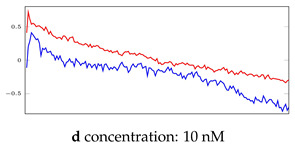	Not Applicable	Not Applicable
Anomaly	Normal	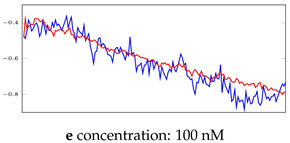	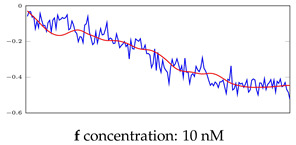	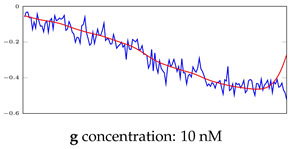
Anomaly	Anomaly	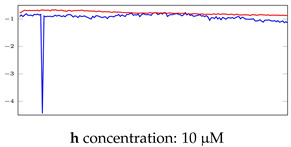	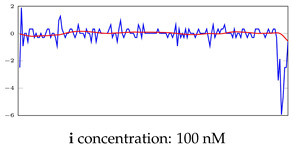	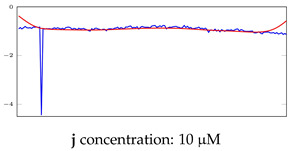

**Table 7:** Visualization of anomaly detection for 35-mer oestradiol dataset. This table shows original and reconstructed segments created by designed autoencoder networks. The blue curves were the original time series, and the red curves were the reconstructed time series.

Actual Class	Predicted Class	Vanilla AE	ULSTM AE	BLSTM AE
Normal	Normal	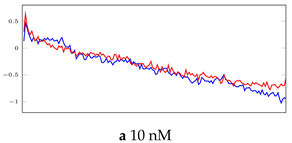	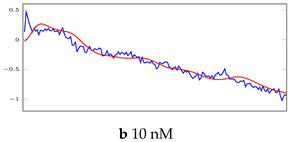	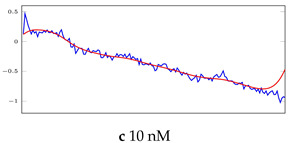
Normal	Anomaly	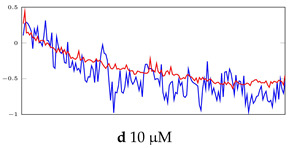	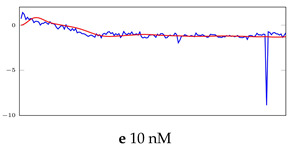	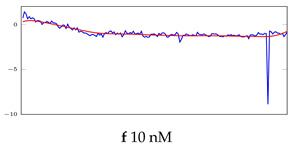
Anomaly	Normal	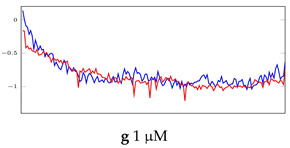	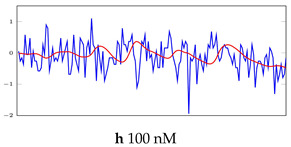	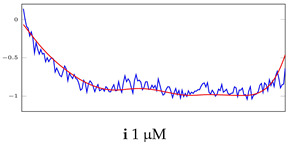
Anomaly	Anomaly	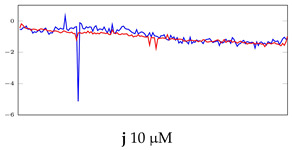	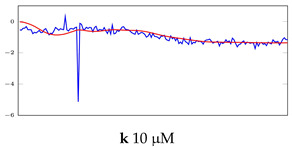	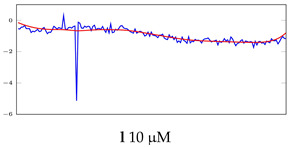

**Table 8:** Representation of anomaly detection in the integrated autoencoder networks.

LSTM Model $\zeta_{1}\left(x\right)$	Vanilla Model $\zeta_{2}\left(x\right)$	Integrated Models ζ(x)
Anomaly (0)	N/A ^1^	Anomaly (0)
Normal (1)	Anomaly (0)	Anomaly (0)
Normal (1)	Normal (1)	Normal (1)

^1^ N/A means that the data was detected as an anomaly in the LSTM network. Thus, it was removed from the sample to the test set that was assessed by the vanilla autoencoder in the next step.

**Table 9:** Confusion Charts for the five proposed models and three datasets.

Dataset	Vanilla AE	ULSTM AE	BLSTM AE	ULSTM & Vanilla AE	BLSTM & Vanilla AE
35-mer Adenosine	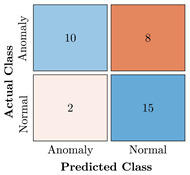	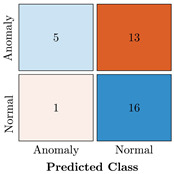	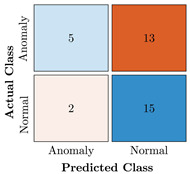	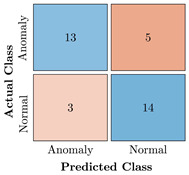	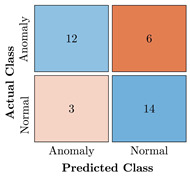
31-mer Oestradiol	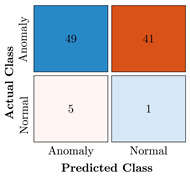	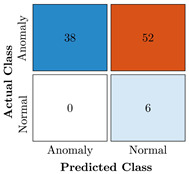	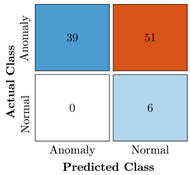	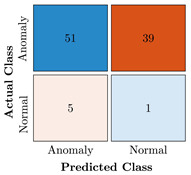	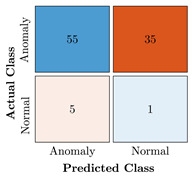
35-mer Oestradiol	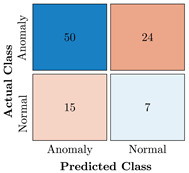	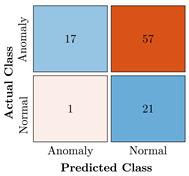	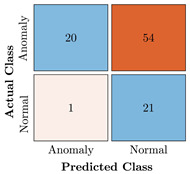	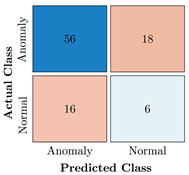	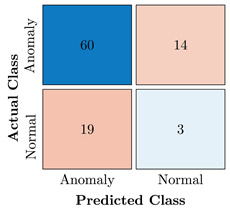

## Data Availability

The data presented in this study might be available on request from the corresponding author. There are restrictions on data availability due to their necessity for our future work.

## Abbreviations

The following abbreviations are used in this manuscript:

ML	Machine Learning
AE	Autoencoder
VAE	Vanilla Autoencoder
ResNet	Residual Network
CNN	Convolution Neural Network
RNN	Recurrent Neural Networks
LSTM	Long Short-Term Memory
AC	Analyte Concentrations
CNT	Carbon Nanotube
FET	Field-Effect Transistor
PDMS	Polydimethylsiloxane
ULSTM	Unidirectional Long Short-Term Memory
BLSTM	Bidirectional Long Short-Term Memory
GRU	Gated Recurrent Unit
